# Acute hydrocephalus caused by a colloid cyst — a case report

**DOI:** 10.1186/s12245-023-00500-5

**Published:** 2023-04-19

**Authors:** Dóra Melicher, Szabolcs Gaál, Tamás Berényi, Bánk Gábor Fenyves, Pál Kaposi Novák, Ambrus Tóth, László Szegedi, Csaba Varga

**Affiliations:** 1 Department of Emergency Medicine, Semmelweis University, Budapest, Hungary; 2 Department of Molecular Biology, Semmelweis University, Budapest, Hungary; 3 Medical Imaging Centre, Semmelweis University, Budapest, Hungary; 4 National Institute of Clinical Neurosciences, Budapest, Hungary

**Keywords:** Colloid cyst, Acute hydrocephalus, Elevated intracranial pressure, Third ventricle

## Abstract

**Background:**

Colloid cysts are rare benign, slowly growing intracranial tumors of endodermal origin. Most colloid cysts are found incidentally and are asymptomatic, but rarely, they can lead to sudden death.

**Case presentation:**

A 73-year-old female patient was admitted to our emergency department with complaints of dizziness, nausea, vomiting, fatigue, walking difficulties, and behavioral changes. CT imaging revealed acute obstructive hydrocephalus attributable to a third ventricular colloid cyst. The patient was immediately transferred to a tertiary center where she underwent successful neurosurgical resection of the mass. Pathology results of the lesion confirmed the diagnosis of colloid cyst.

**Conclusion:**

The case we present emphasizes the critical importance of prompt identification of warning signs, complex thinking, and evaluation. Establishing the right diagnostic approach early on can facilitate accurate diagnosis.

## Background

Colloid cysts (CCs) are benign growths considered as rare developmental malformation. They are composed of an outer fibrous layer and an inner epithelium of ciliated or mucin-producing cells [1–3]. CCs account for ~ 2% of primary brain tumors and are located at the roof of the third ventricle adjacent to the foramina of Monro in 99% of the cases [4–6].

Most colloid cysts are found incidentally on brain imaging and are asymptomatic. CCs tend to slowly increase in size over time, and although most may never reach a size that will cause any problem, some may grow more quickly and become symptomatic, even lead to sudden death due to causing acute hydrocephalus, raised intracranial pressure (ICP), or hypothalamic stimulation [7–11]. Obstructive hydrocephalus is precipitated by blockage of cerebrospinal fluid (CSF) outflow from the lateral ventricles at the foramen of Monro [3, 12].

Surgical treatment of colloid cysts include craniotomy with excision via transcallosal or transcortical route, endoscopic removal, or stereotactic aspiration, and external ventricular drains could also be performed as bridge therapy in life-threatening hydrocephalus [3]. The management of asymptomatic cases is influenced by the lesion size, the presence of hydrocephalus, the patient’s age and preference, and associated medical conditions [8, 10, 13]. For patients without hydrocephalus and a colloid cyst of usually < 10 mm in diameter, follow-up is advised [14, 15].

## Case presentation

A 73-year-old female patient was brought to our emergency department (Department of Emergency Medicine, Semmelweis University) by ambulance, complaining of worsening dizziness, nausea, vomiting, fatigue, and walking difficulties. Her past medical history included hypertension and colorectal carcinoma that were treated surgically and with chemotherapy 20 years ago. She reported taking antihypertensive medications only. On further questioning, the patient specifically denied any history of neurological problems.

On inquiring about the details including the timing of her complaints, she mentioned being dizzy in the last 3 weeks. She described it as rather feeling faint or light-headed as opposed to spinning sensation. She could not clearly establish whether the dizziness worsened to any positional change and reported that her unsteadiness was slowly progressing over the last 2 weeks. The emergency physician (EP) contacted his son by phone, whereby the relatives described the patient as being generally slow, forgetful, and hesitant during conversations, more noticeably in the previous 3 days, and decided to call the ambulance because they noticed as if she shortly lost consciousness while sitting in the chair. The family members also confirmed that her past medical history was significant only for hypertension and colorectal carcinoma 20 years ago.

Upon arrival, her blood pressure was 134/64 Hgmm, heart rate 54/min, respiratory rate 18/min, SpO2 95%, temperature 37.0 °C, and blood glucose was 7.4 mmol/L. The patient had an unremarkable physical exam except for slightly slowed psychomotor pace and slightly drier mucus membranes. No cranial nerve deficits were found. Arterial blood gas detected hypoxia (PaO2: 56.5 Hgmm) and lactate level of 1.2 mmol/L. Lab work included complete blood count and basic metabolic panel which were unremarkable.

**Fig. 1:**
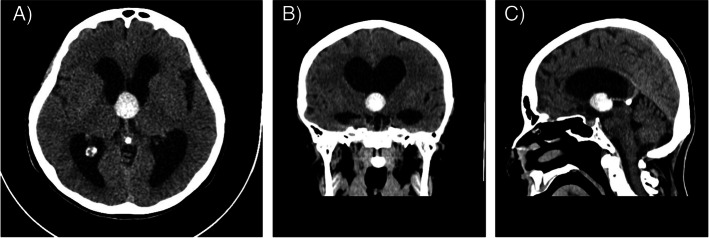
Colloid cyst (initial images). Unenhanced CT images show a well-defined hyperattenuating lesion on the anterior roof of the third ventricle at the foramina of Monro causing obstructive hydrocephalus. (**A**) axial, (**B**) coronal (**C**) sagittal reconstruction

The progressing and alarming nature of the patient’s symptoms and the family’s note about altered behavior prompted a non-contrast computed tomography (CT) of the head in the ED. The cranial CT revealed acute obstructive hydrocephalus with significant dilation of the lateral ventricles (Evans index: 0.45) caused by a colloid cyst. The cyst was described as a 21 × 22 mm hyperdense mass in the roof region of the third ventricle, significantly impairing liquor passage (Fig. 1/A, B, C). After imaging, neurology consultation was requested, which noted horizontal gaze nystagmus (that was not present upon arrival). The neurologist also noted disorientation in space, slightly slowed psychomotor pace, mild truncal ataxia, slight gait ataxia, positive palmomental reflex on both sides, and intact cranial nerves.

Based on the presenting symptoms, the CT findings, and the neurological assessment, we consulted the on-call neurosurgeon promptly. As urgent surgical treatment was recommended, the patient was immediately transferred to a tertiary neurosurgical center.

**Fig. 2:**
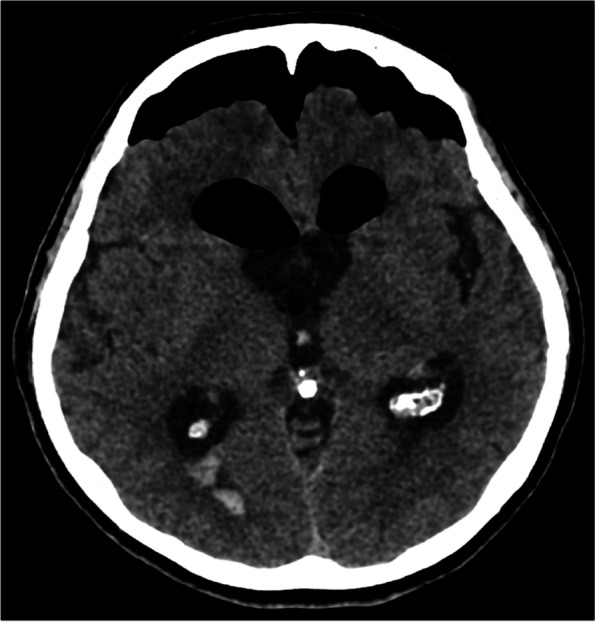
Axial CT image right after surgical resection of the colloid cyst shows pneumocephalus and small amount of intraventricular hemorrhage which can be considered normal postoperative findings. The hydrocephalus has not yet resolved

**Fig. 3:**
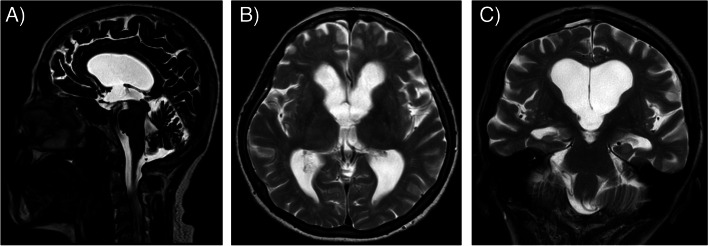
MRI images 4 months after surgical resection. T2-weighted sagittal TSE DRIVE (**A**), T2-weighted axial (**B**) and coronal (**C**) MRI images demonstrate a postoperative defect on the roof of the third ventricle. There is moderate residual hydrocephalus with no signs of obstruction. TSE: turbo spin-echo

The patient underwent neurosurgical resection of the mass carried out through a right frontal craniotomy (Fig. 2). Sample from the mass was sent for pathology, which later confirmed results consistent with a colloid cyst. On the fifth postoperative day, the patient was stable and transferred to the neurology department of a town hospital for further secondary care. She was discharged home 10 days later. 4 months after surgery MRI showed no signs of obstruction (Fig. 3/A, B, C).

## Discussion

Despite their rare occurrence and benign character, colloid cysts are of great clinical significance as they can cause acute deterioration and sudden death due to their critical location [7–11]. On the one hand, acute decompensation can result from acute severe increase in ICP due to intracystic hemorrhage or acute obstruction of the foramen of Monro and in the absence of immediate surgical intervention, brain herniation. On the other hand, acute decompensation was also found to occur without significant hydrocephalus due to compression of the hypothalamus, which in the absence of immediate surgical intervention can lead to autonomic dysfunction causing sudden cardiac failure [11, 16].

In our case report, we discuss both the path that led us to establishing the optimal diagnostic approach early on and also the potential pitfalls that can lead to missed or delayed diagnosis in ED settings.

Emergency physicians have to be trained to promptly identify the warning signs of potentially life-threatening medical emergencies. They should look for clinical indicators (“red flags”) of possible serious underling conditions. Clinical suspicion for raised intracranial pressure should be raised if a patient presents with headache, vomiting, altered mental state, and visual changes [17]. Causes of raised intracranial pressure include neoplasms, stroke, traumatic hematomas, abscess, encephalitis, meningitis, diffuse head injury, seizures, encephalopathy (hepatic, toxic, uremic, or septic), hypoxemic-ischemic encephalopathy, water intoxication, Reye’s syndrome, obstruction to major venous sinuses, vascular malformations, and disturbances of CSF circulation owing to obstructive hydrocephalus, communicating hydrocephalus, or subarachnoid hemorrhage [18].

Common symptoms of acute hydrocephalus can be headache, nausea, vomiting, lethargy, short-term memory loss, unsteady gait, ataxia, failure of upward gaze, papilloedema, and increased reflexes, while slowly progressive hydrocephalus could be characterized with more subtle findings such as generalized weakness, urinary incontinence, walking difficulties, falls, behavioral changes, and memory deficits [3, 12, 15]. The classical triad of symptoms typically present with hydrocephalus is gait abnormality, cognitive disturbance, and urinary incontinence [19], which however may not occur all at the same time, suggesting that gait disturbance with one additional feature can give ground to consider the diagnosis [20].

In our case, the diagnostic challenge was that mild ataxia/walking difficulty was not accompanied with neither urinary incontinence nor headache. Also, the patient was initially oriented and gave adequate answers. The only alarming sign of altered behavior was the somewhat slower verbalization and decision-making, but without prior knowledge of her baseline mental state, it was hard to interpret correctly. Only the collateral history given by the family members gave valuable help to the EP to consider the findings as altered mental state. Here, we highlight the importance of targeted questioning of relatives since although their answers could be valuable, the conversation should not delay the optimal time frame of ED physical examinations.

Importantly, our patient did not present with headache, which is the most common symptom of CCs (present in 68–100% of patients) [6, 21–24]. Hence, a constellation of acute headache plus associated nausea/vomiting or neurological sign (obvious red flags) was also absent in our case.

Furthermore, we detail potential pitfalls related to our specific case. Upon arrival to our ED, the patient was assigned to level 3 (urgent) according to the Hungarian Emergency Triage System (MSTR) [25]. The triage nurse indicated a note stating “positional vertigo” that proved to be incorrect after further evaluation of the EP. The main complaint of our patient was dizziness, which when further interrogated by the EP, she described as rather feeling faint or light-headed as opposed to spinning sensation. The patient could not clearly establish whether the dizziness worsened to any positional change and reported experiencing it for 3 weeks. Our case again highlights the importance to differentiate vertigo-like symptoms from other forms of dizziness, light-headedness, or imbalance and distinguish central from peripheral causes of vertigo, as establishing early on the right diagnostic approach that can facilitate an accurate diagnosis is essential in life-threatening causes [26, 27].

We would also like to remark the possible trap of attributing the patient’s complaints mostly to extreme weather conditions or lack of sufficient water drinking, since on the day of her visit, just as in the previous 2 weeks, the weather was particularly hot (outdoor temperature 35 °C) and the patient indeed showed signs of dehydration.

We would also like to call attention to the importance of prevention. Considering the increasing availability of current imaging techniques in various clinical practices, CCs are highly likely to be coincidentally diagnosed before growing in size and leading to serious complications such as acute obstructive hydrocephalus. In case of incidental findings, patient education about alarming symptoms besides regular clinical and radiological follow-up are critical for overcoming potential lethal consequences of acute progression. We would also like to highlight that as detailed above, emerging literature shows that a number of mechanisms could explain the causes of acute conditions and sudden death due to colloid cysts. In view of that, sudden deterioration, life-threatening complications, and death should be discussed with all patients despite the cyst size. A recent review of 65 cases of colloid cyst attributed deaths concluded that operative management should be recommended for CCs above 10 mm regardless of presence or absence of symptoms [11]. Patients kept on observation should be thoroughly instructed to report to the nearest emergency department if develop severe headache or vomiting or prominent walking difficulties and unsteadiness, while close family members should also be aware of alarming signals and the importance of mentioning any earlier diagnosis.

## Conclusion

The present case emphasizes the critical role emergency medicine physicians have in promptly identifying the warning signs that could underly life-threatening emergencies such as acute hydrocephalus. Establishing early on the right diagnostic approach can facilitate the accurate diagnosis. Using complex thinking and evaluation, based on thorough questioning, history, heteroanamnesis, and targeted physical examination, constitutes key elements of this process. In the presented case, early identification for a need to order urgent imaging imminently revealed the acute complications caused by a rare colloid cyst. Should such acute cases occur, in order to prevent life-threatening consequences and death, prompt intervention is crucial, as optimal treatment of critical conditions caused by colloid cysts carries excellent prognosis.

## Data Availability

Not applicable.

## Abbreviations

CCs: Colloid cysts
CSF: Cerebrospinal fluid
EP: Emergency medicine physician
ICP: Intracranial pressure
TSE: Turbo spin echo
